# Can Hormonal Therapy Improve the Outcomes of mTESE in Patients With Non‐Obstructive Azoospermia?

**DOI:** 10.1111/andr.70292

**Published:** 2026-06-25

**Authors:** Mattia Anfosso, Maria Schubert, Jann‐Frederik Cremers, Sabine Kliesch, Michael Zitzmann, Simone Bier

**Affiliations:** ^1^ Department of Andrology Centre of Reproductive Medicine and Andrology (CeRA) University Hospital of Münster Münster Germany

**Keywords:** hormonal therapy, hypogonadism, microdissection testicular sperm extraction (mTESE), non-obstructive azoospermia, sperm retrieval rate

## Abstract

**Background:**

Non‐obstructive azoospermia (NOA) represents the most severe form of male infertility. Hypogonadism is common in NOA patients, and normal testosterone (T) levels are considered essential for spermatogenesis. Fertility‐preserving hormonal therapy (FpHT) has been proposed to optimize hormonal milieu and improve sperm retrieval rates (SRR) at microdissection testicular sperm extraction (mTESE), although evidence remains controversial and meta‐analyses have not shown consistent benefit.

**Aims:**

To determine whether FpHT improves SRR in hypogonadal men with NOA undergoing mTESE.

**Methods:**

We retrospectively evaluated 1256 selected men with NOA (601 Klinefelter syndrome [KS], 655 normal karyotype) from a cohort of 3086 men who underwent mTESE at our tertiary andrology center between 2010 and 2024. Patients with obstructive azoospermia, prior gonadotoxic exposure, or hypogonadotropic hypogonadism were excluded. All participants underwent comprehensive clinical, hormonal, and genetic assessment. Comparisons were performed between eugonadal and hypogonadal patients, and within the hypogonadal group, between those who received FpHT and those who did not.

**Results:**

Hypogonadism was present in 49.9% of patients, predominantly in KS (*p* < 0.001). FpHT significantly increased serum T levels in both groups (ΔT +3.1 nmol/L in KS; +5.6 nmol/L in 46, XY; *p* < 0.001). However, despite biochemical normalization, FpHT did not improve SRR in either karyotypic group (overall SRR: 32.4%). Multivariate analysis identified younger age as the sole independent predictor of successful retrieval in KS (*p* = 0.011).

**Conclusion:**

FpHT restores serum androgen levels but does not improve spermatogenic activity or sperm retrieval rates in men with NOA. These findings support current EAU guidelines and indicate that FpHT should be used to treat symptomatic hypogonadism rather than to enhance fertility outcomes before mTESE.

## 1 Background

*Non‐obstructive azoospermia (NOA)* refers to the absence of sperm in the ejaculate due to severely reduced or absent production of mature spermatozoa [1, 2]. Considered one of the most severe forms of male infertility [3], NOA is estimated to affect approximately 1% of the general male population and accounts for about 15%–20% of cases among infertile men.

It is a clinical condition resulting from a variety of pathological processes that impact spermatogenesis at different stages. The underlying cause can be identified in only 30% [4] of cases and includes: *genetic factors* [5, 6, 7] (e.g., Klinefelter syndrome, AZF microdeletions), oncological conditions [8] (e.g., testicular cancer, pituitary adenomas), *iatrogenic causes* [9, 10] (e.g., chemo/radiotherapy, surgical procedures or use of exogenous testosterone), traumatic *injuries*, and *infections* [2] (e.g., mumps orchitis). Representing the remaining 61%, *idiopathic forms*—that is, cases without an identifiable cause—are thought to result from a multifactorial process involving genetic background, lifestyle, and environmental factors [2, 11].

Initially described by Schlegel et al. in 1999 [12], *microdissection testicular sperm extraction (mTESE)* has since become widely accepted as the technique of choice for patients with NOA pursuing biological parenthood [12, 13, 14, 15]. The procedure involves the microsurgical dissection of testicular tissue in order to identify mature spermatozoa suitable for use in intracytoplasmic sperm injection (ICSI) [16, 17]. Surgical sperm retrieval rates (SRR) reported in the literature are heterogeneous, ranging from 40% to 60% [14, 15, 18], with significantly poorer outcomes observed in cases of idiopathic NOA [16].

Hypogonadism—defined as serum total testosterone (T) < 12 nmol/L confirmed on two separate morning measurements—is common among men with NOA [19] and historically, serum T concentrations have been proposed as a potential predictor of surgical outcomes, with lower T levels correlating with reduced SRR. This hypothesis is based on the rationale that reductions greater than 75% in intratesticular T levels have been associated with spermatogenic failure in murine models [20, 21, 22]. Consequently, fertility‐preserving hormonal therapy (FpHT)—including gonadotropins, selective estrogen receptor modulators (SERMs), and aromatase inhibitors—has been widely adopted in clinical practice with the aim of increasing endogenous T production, optimizing the hormonal milieu, and potentially enhancing mTESE outcomes [3, 23, 24, 25].

Despite its theoretical appeal and widespread use, the clinical efficacy of FpHT remains controversial. Recent meta‐analyses have failed to demonstrate a consistent benefit of preoperative hormonal optimization on SRR [26, 27], with potential improvements limited to highly selected subgroups such as men with hypogonadotropic hypogonadism or normogonadotropic hypogonadism [20, 27]. Critically, many earlier studies included heterogeneous patient populations—potentially mixing subclinical obstructive cases with true NOA—or lacked adequate control groups, limiting the validity of their conclusions [20, 23]. Moreover, current European Association of Urology (EAU) guidelines do not recommend routine FpHT prior to mTESE due to insufficient evidence [28]. Yet, in clinical practice, FpHT continues to be prescribed frequently, driven by the hope of improving fertility outcomes in a patient population with limited therapeutic options.

The gap between current guidelines and widespread clinical use highlights an unmet need for high‐quality evidence based on large patient cohorts with rigorous inclusion criteria, which may help define the true role of FpHT in the management of hypogonadal men with NOA.

The present study aims to evaluate whether FpHT improves SRR in men with NOA and concomitant hypogonadism undergoing mTESE.

## 2 Patients and Methods

### 2.1 Study Population

In this retrospective study we analyzed mTESE results in respect to karyotype, presurgical therapy, in 1256 from 3086 patients with NOA who underwent mTESE at our Department of Andrology, Centre of Reproductive Medicine and Andrology (CeRA) between January 2010 and January 2024. The study was designed and reported in accordance with the Strengthening the Reporting of Observational Studies in Epidemiology (STROBE) guidelines [29]. The study was approved by the local ethical committee (Ethical Committee Westfalen‐Lippe, Münster, 2025‐945‐f‐S).

**FIGURE 1:**
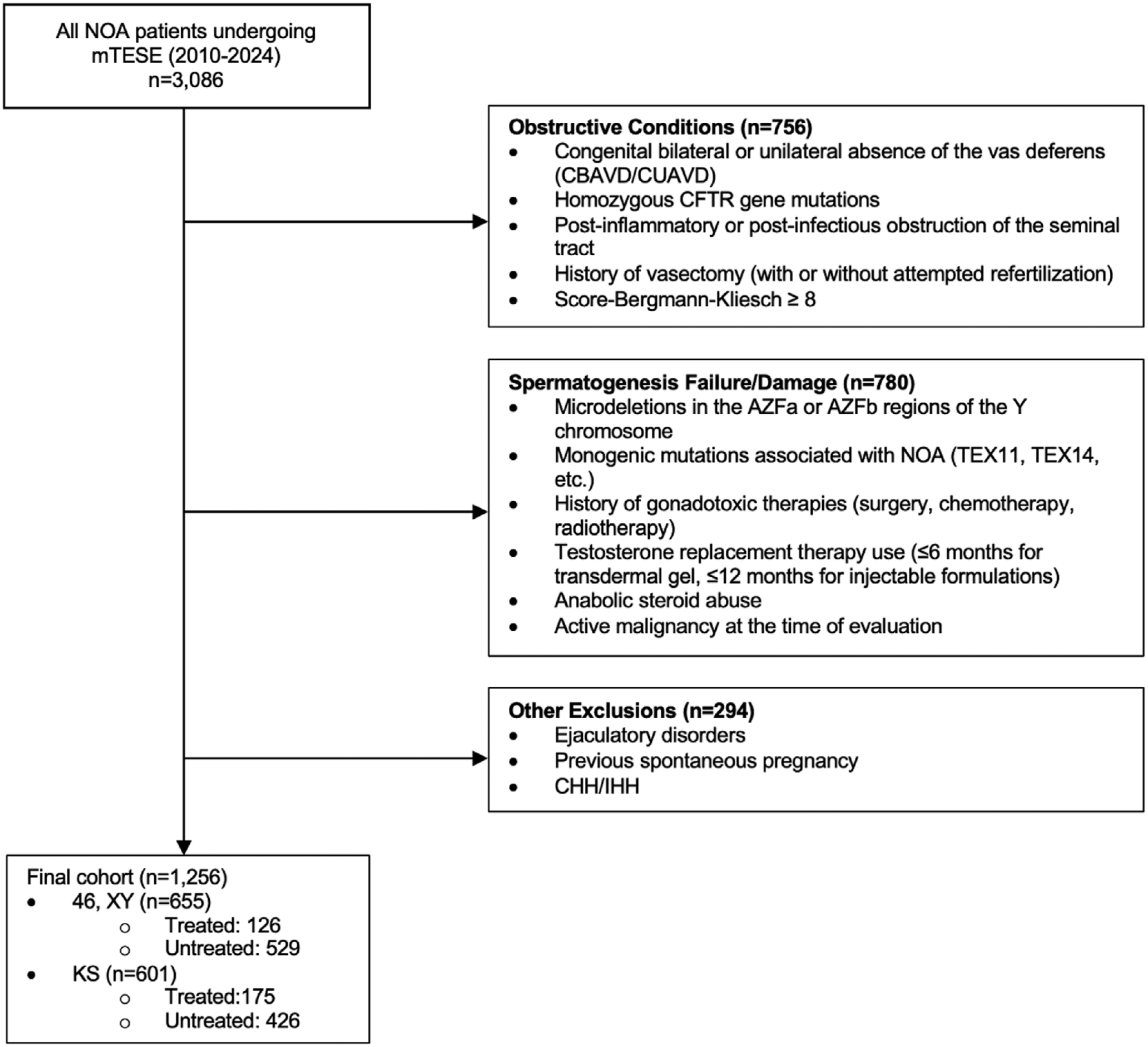
Patient selection flowchart (2010–2024).

Patients were included regardless of age, provided that at least Tanner stage 3–4 had been reached. Comparisons were performed between eugonadal and hypogonadal patients, and within the hypogonadal group, between those who received FpHT and those who did not. Results were stratified by age to evaluate potential age‐related influences on therapy outcomes and descriptive surgical parameters. To minimize confounding factors, patients with obstructive conditions were excluded, as these are associated with higher SRR independent of hormonal treatment. Similarly, conditions causing spermatogenesis failure or severe germ cell damage were excluded due to their irreversible nature. Finally, CHH/IHH—the condition most likely to respond favorably to FpHT—was excluded to avoid positive selection bias. The exclusion criteria are summarized in Figure 1.

All patients underwent a comprehensive diagnostic workup prior to surgical intervention: medical history and clinical evaluation, including somatic parameters, ultrasound of the testes, hormonal profiling, semen analysis according to WHO criteria, and genetic testing.

### 2.2 Data Input and Extraction

Data were extracted from our institutional database (Androbase) [30]. For each patient, medical records were reviewed from the time of their first presentation up to the date of the surgical procedure. Collected information included patient demographics and clinical characteristics (age, body mass index [BMI], and smoking status), testicular findings (testicular volume, history of cryptorchidism, testicular microlithiasis, and presence of varicocele), hormonal levels (follicle‐stimulating hormone [FSH], luteinizing hormone [LH], total and free testosterone [T and fT]), genetic data (karyotype, Y‐chromosomal microdeletions, CFTR mutations, and single nucleotide polymorphisms [SNPs] in *FSHB* and *FSHR* genes), and relevant comorbidities (arterial hypertension, type I and II diabetes mellitus [DMI or DMII], hypothyroidism, and history of inguinal hernia). For patients who underwent medical treatment, the specific type of medication as well as the duration of therapy, were recorded and included in the analysis. Postoperative hormone levels and data on complications were excluded from the analysis due to a high proportion of missing values.

### 2.3 Testicular Ultrasound

Testicular ultrasound was performed using a high‐frequency linear transducer (12–15 MHz, Sony Affiniti 70G PureWave) to assess according to the clinical standard parenchymal homogeneity, echogenicity, and paratesticular structures, contributing to the evaluation of potential obstructive causes and to the exclusion of testicular tumors [31]. For testicular volume measurement, a high‐frequency convex transducer was used, applying the ellipsoid formula [32]. For statistical purposes, total bitesticular volume (hereafter referred to as “total testicular volume” or TV) was used, except in cases of unilateral agenesis, atrophy, or previous orchiectomy, where the volume of the remaining functional testis was considered.

### 2.4 Genetic Analysis and SNP Genotyping

Karyotype analysis was performed in all patients. Screening for Y‐chromosomal microdeletions (AZF region), was conducted only in cases with a normal karyotype. CFTR gene mutation analysis was only performed in suspected obstructive azoospermia and a normal karyotype. For genotyping of the *FSHB* and *FSHR* genes, genomic DNA was extracted from EDTA‐anticoagulated blood using either the FlexiGene DNA Kit (QIAGEN, Düsseldorf, Germany) or the DNA Extract All Kit (Applied Biosystems, Darmstadt, Germany) and was analyzed in the frame of previous research projects as published earlier [33, 34, 35].

### 2.5 Hormone Assessment

Hormonal evaluation, including FSH, LH, T and fT, was performed as part of the standard infertility workup. Patients who had previously received testosterone supplementation had discontinued treatment at least 6–12 months before undergoing mTESE (depending on the type of medication), and pituitary function recovery was monitored by measuring gonadotropin levels.

In patients who received FpHT, two hormone profiles were included in the statistical analysis: the last available baseline measurement obtained prior to treatment initiation, and the most recent pre‐surgical value under active therapy, collected within 3 months before surgery. For patients who did not undergo FpHT, only the last available hormonal assessment within the 3‐month presurgical window was considered.

All venous blood samples were collected between 8:00 a.m. and 12:00 p.m. Serum or plasma was separated by centrifugation at 800 g. Samples were either analyzed immediately or snap frozen and stored at ‐20°C until analysis. Serum testosterone concentrations were measured using a commercial ELISA kit (DRG Instruments, Marburg, Germany). This immunoassay is calibrated quarterly against liquid chromatography–mass spectrometry (LC–MS) standards; the assay regularly passes quality control and yields testosterone values with <10% variation in the 5–20 nmol/L range. Intra‐assay coefficients of variation (CVs) were <2%, and inter‐assay CVs were <5% on average.

Serum LH and FSH concentrations were measured using highly specific time‐resolved fluoroimmunoassays (Autodelfia, Freiburg, Germany). The mean intra‐assay CVs were <2%, and mean inter‐assay CVs were <5%. All hormone assays were also subject to blinded external quality controls on a quarterly basis, which were consistently passed. Reference ranges were defined as follows: FSH (1–7.0 U/L), LH (2–10 U/L), total testosterone (>12 nmol/L), and free testosterone (>250 pmol/L).

### 2.6 Hypogonadism: Definition and Hormonal Stimulation

Diagnosis of hypogonadism followed guideline‐based clinical practice applicable during the study period. Biochemical hypogonadism was defined as total serum testosterone concentrations below 12 nmol/L or calculated free testosterone concentrations below 250 pmol/L on at least two separate measurements [36, 37]. The threshold for calculated free testosterone was based on the ISA/ISSAM/EAU recommendations by Nieschlag et al., in which free testosterone concentrations above 250 pmol/L were considered unlikely to require testosterone substitution. Since our clinic has continued to use the same testosterone assay and the same method for calculating free testosterone, this threshold has been retained in routine practice for reasons of methodological continuity, comparability between patients, and assessment of intra‐individual changes during treatment. In all cases, biochemical criteria had to be accompanied by at least two symptoms suggestive of testosterone deficiency.

According to the associated gonadotropin levels, patients were classified as having primary hypogonadism, in the presence of elevated gonadotropins, or functional hypogonadism, when gonadotropin levels were within the normal range. Patients with secondary hypogonadism, defined by low gonadotropin levels, were not included in the study.

Hormonal stimulation with FpHT was subsequently offered to all patients with biochemical hypogonadism, regardless of karyotype. Specifically, in cases of more severe hypogonadism (T < 8 nmol/L), the proposed protocol generally included the administration of human chorionic gonadotropin (hCG), starting at a dose of 1500 IU subcutaneously twice per week, with the possibility of dose escalation depending on clinical and hormonal response. Treatment was intended to last for at least 6 months, with follow‐up visits scheduled every 3 months. In the event of marked suppression of FSH levels during therapy, recombinant FSH (follitropin alfa, e.g., Gonal‐F) was added at a dose of 150 IU subcutaneously three times per week.

In cases of milder biochemical hypogonadism (T > 8 nmol/L and < 12 nmol/L), primary treatment options included oral aromatase inhibitors such as anastrozole at 1 mg daily for at least three months especially in adipose patients, or selective estrogen receptor modulators (SERMs), such as tamoxifen at 20 mg daily or clomiphene citrate at 25–50 mg daily, also for a minimum duration of 3 months.

The primary goal of FpHT was to achieve eugonadal testosterone levels or, alternatively, to maintain or reach T values ≥ 8 nmol/L, a threshold below which SRR was shown to be strongly reduced in a previously published study from our center [24].

Because all aforementioned medications are used off‐label for male eugonadotropic or hypergonadotropic infertility, the treatment options, associated costs, and potential side effects were thoroughly discussed with each patient before initiating therapy.

### 2.7 Sperm Retrieval

mTESE was performed by experienced urologists following a modified technique first described by Kliesch [24]. The surgery was carried out under general or regional anesthesia with the patient in the supine position. A midline scrotal incision (∼3 cm) was made to expose the testis, followed by a lateral semilunar incision (∼3 cm) in the tunica albuginea, parallel to the epididymis. The tunica was then elevated to allow full visualization of the testicular parenchyma. Using a surgical microscope (Zeiss OpmiVario S88 and Zeiss Tivato 700, Jena, Germany,) with 5× to 25× magnification, the testicular tissue was carefully examined segment by segment—both superficially and in depth—to identify the most prominent and dilated seminiferous tubules. A small indicator fragment from each biopsy—typically eight to sixteen samples collected from both testes—was set aside for subsequent sperm analysis. Testicular specimens were individually and meticulously processed in the in vitro fertilization (IVF) laboratory. Sperm retrieval was considered successful (positive SRR) if at least one spermatozoon was identified. In cases where no sperm were initially observed, enzymatic digestion of the testicular suspension was discussed with the patient and subsequently performed, when agreed upon.

### 2.8 Testicular Histopathology

Bilateral testicular biopsies (unilateral in cases of atrophy, agenesis or orchidectomy) were performed during mTESE, with samples taken from at least two different distinct regions of each testis. For histological evaluation, fragments approximately 5 mm in length and 2 mm in diameter were fixed in Bouin's solution, embedded in paraffin, sectioned, and stained with hematoxylin‐eosin and periodic acid–Schiff. All seminiferous tubules were assessed individually. Classification was based on the predominant histological pattern: presence of hypospermatogenesis (defined by elongated spermatids), maturation arrest (MA, spermatogonial or meiotic), Sertoli cell‐only syndrome (SCO) or tubular shadows (TS). In cases where spermatozoa were retrieved during mTESE from the same region as the biopsy, the corresponding specimen was classified as showing complete spermatogenesis [38].

Histological evaluation was complemented by the Bergmann–Kliesch score [36, 37], a semiquantitative histological index that describes the degree of spermatogenesis in a testicular biopsy sample by estimating the percentage of seminiferous tubules containing elongated spermatids and thus the degree of spermatogenic completeness. Score 10 reflects 100% of tubules presenting with elongated spermatids. Scores below 8 are considered indicative of a qualitatively and quantitatively impaired spermatogenesis, typical of the most severe forms of NOA and associated with low probability of sperm retrieval, whereas scores equal to or greater than 8 denote qualitatively normal spermatogenesis and are more compatible with obstruction.

### 2.9 Outcomes

The primary outcome of the study was the presence or absence of sperm retrieval during mTESE. Secondary outcomes included pre‐ and post‐treatment hormonal levels, as well as histological findings.

### 2.10 Patient Subgroups

For the purpose of the analyses, the aforementioned outcomes were compared across clinically relevant subcategories. The study population was initially divided into two main groups according to karyotype, and subsequently stratified into three subgroups based on T levels and FpHT administration: untreated eugonadal, untreated hypogonadal, and FpHT‐treated hypogonadal patients.

Additional stratifications and secondary analyses were conducted according to T levels (<8 nmol/L, 8–12 nmol/L, and >12 nmol/L) and histological patterns (TS, SCO, MA, and hypospermatogenesis). Within the hypogonadal subgroup, comparisons were performed based on gonadotropin levels (hyper‐ vs. normogonadotropic), while within the FpHT‐treated subgroup, analyses were conducted according to the type of therapy administered.

### 2.11 Statistical Analysis

Statistical analysis was performed using R software (version 2024.12.1+563). The normality of variable distribution was assessed using the Shapiro–Wilk test. Categorical variables were described using absolute frequencies and percentages, while continuous variables were summarized using the median and interquartile range (IQR), in cases of non‐normal distribution.

Univariate analysis: Comparisons between categorical variables (e.g., therapy vs. no therapy) were performed using the Chi‐square test or Fisher's exact test, as appropriate. For continuous variables, either Student's *t*‐test or the Mann–Whitney *U* test was used, depending on data distribution.

Multivariate analysis (Logistic regression): Multivariate analysis was conducted using logistic regression, with sperm retrieval outcome (positive/negative) as the dependent variable. In the total patient cohort, the following independent variables were included in the model: age, BMI, testicular volume, presence of comorbidities, hormonal levels at baseline (FSH, LH, total and free testosterone levels), and the FSHB c.‐211 polymorphism. In the subgroup of treated patients, baseline hormonal values were replaced by post‐treatment levels, and hormonal therapy (yes/no) was included as a variable.

Collinearity among predictors was assessed using the variance inflation factor (VIF), and possible interaction effects (e.g., testosterone and treatment type) were also explored. Results are reported as odds ratios (ORs) with corresponding 95% confidence intervals and *p*‐values.

## 3 Results

### 3.1 Clinical Characteristics of Patient Cohort

Among the 3086 patients who underwent mTESE at our center, 1256 men met the inclusion criteria, comprising 601 (48.8%) patients with KS and 655 (51.2%) with a normal karyotype. Table 1 summarizes the baseline characteristics of the total study population. The overall SRR was 32.4%, and did not differ significantly when stratified by karyotype (*p* = 0.1).

**TABLE 1:** Baseline characteristics and sperm retrieval rates of the entire cohort who underwent Micro‐TESE, stratified by karyotype (46, XY vs. 47, XXY).

	All patients	46, XY	KS	*p*
Total patients, *n* (%)	1256	655 (51.2)	601 (48.8)	
Hypergonadotropic azoospermia, *n* (%)	1177 (93.8)	581 (88.7)	596 (99.2)	**<0.0001** ^c^
Normogonadotropic azoospermia, *n* (%)	79 (6.3)	74 (11.3)	5 (0.8)	**<0.0001** ^c^
Age (years), median (IQR)	31 (19–36)	35 (31–39)	19 (15–30)	**<0.0001** ^a^
BMI (kg/m^2^), median (IQR)	25.3 (22.2–29)	26.8 (24.2–30.2)	23.4 (19.6–26.9)	**<0.0001** ^a^
Smoker, *n* (%)	358 (28.5)	211 (32.2)	147 (24.4)	**<0.0001** ^c^
Total TV (mL), median (IQR)	8(4‐20)	19 (12–26)	4 (2–5)	**<0.0001** ^a^
**Comorbidities, *n* (%)**				
Idiopathic NOA	351 (28.0)	351 (53.6)	—	
History of undescended testis	295 (23.5)	167 (13.3)	128 (21.3)	0.14^c^
Varicocele	145 (11.6)	102 (8.1)	43 (7.2)	**<0.0001** ^c^
AZFc microdeletions	31 (2.5)	31 (4.7)	—	
Inguinal hernia	63 (5.0)	46 (3.7)	17 (2.8)	**0.01** ^c^
Diabetes mellitus type 1	9 (0.7)	8 (1.2)	1(0.2)	**<0.05** ^b^
Diabetes mellitus type 2	8 (0.6)	7 (1.1)	1(0.2)	0.07^b^
Arterial hypertension	40 (3.2)	32(4.9)	8(1.3)	**<0.001** ^c^
Hypothyreosis	57 (4.5)	45(6.9)	12(2)	**<0.0001** ^c^
Hypogonadism (total)	627 (49.9)	243 (37.1)	384 (63.9)	**<0.0001** ^c^
Treated	301 (23.9)	126 (19.2)	175 (29.1)	0.16^a^
**Baseline hormonal level, median (IQR)**				
FSH (IU/L)	24.1 (14.8‐34.2)	18 (10.9–26.3)	30.7 (22.8–41.4)	**<0.0001** ^a^
LH (IU/L)	9.1 (5.2‐14.7)	6 (4–8.8)	14.2 (10.2–18.6)	**<0.0001** ^a^
T (nmol/L)	12.2 (8.7‐16.6)	13.8 (10–18.5)	10.5 (7.5–14.6)	**<0.0001** ^a^
fT (pmol/L)	249 (180‐335)	289 (218–368)	206 (147.8–285)	**<0.0001** ^a^
**Positive sperm retrieval, *n* (%)**	407 (32.4)	226 (34.6)	181 (30.1)	0.1^c^

*Note*: The table compares demographic features, comorbidities, hormonal profiles, and surgical outcomes between patients with normal karyotype and Klinefelter syndrome.

Abbreviations: BMI, body mass index; FSH, follicle‐stimulating hormone; fT, free testosterone; KS, Klinefelter syndrome; LH, luteinizing hormone; T, total testosterone; TV, testicular volume.

^a^ Mann–Whitney test.

^b^ Fisher's test.

^c^ Chi‐squared test.

Patients with KS were significantly younger than those with a normal karyotype (*p* < 0.001), had a lower BMI (*p* < 0.001), a lower prevalence of smoking (*p* < 0.001), and fewer comorbidities, including inguinal hernia, arterial hypertension, hypothyroidism, and varicocele (all *p* < 0.05). The prevalence of undescended testis was comparable between the two groups (*p* = 0.14), whereas the median total testicular volume was significantly lower in KS patients (*p* < 0.001).

Hypogonadism was identified in 627 patients (49.9%), occurring significantly more frequently in KS than in 46, XY men (384 vs. 243; *p* < 0.001), and was treated in 301 of these cases (126 with 46, XY karyotype and 175 with KS; *p* = 0.16).

Hormonal levels differed significantly between the two cohorts, showing KS patients higher FSH and LH levels (*p* < 0.0001), along with lower T and fT concentrations (*p* < 0.0001), in comparison with 46, XY patients.

Histological analysis shown in Table 4 revealed a poorer qualitative pattern in patients with KS, characterized by a significantly higher prevalence of TS and SCO and a lower incidence of hypospermatogenesis (*p* < 0.001), while no difference was observed in the rate of MA (*p* = 0.95).

### 3.2 Hypogonadism: Treated versus untreated

Few significant differences emerged from the comparison between FpHT‐treated and untreated hypogonadal patient cohorts (Table 2). Untreated individuals showed lower BMI and larger testicular volume (both *p* < 0.01), regardless of karyotype. Among KS patients, untreated men were significantly younger and less likely to be smokers (both *p* ≤ 0.01). In both karyotype groups, T and fT levels were lower in the treated cohorts (both *p* < 0.001).

**TABLE 2:** Comparison of baseline characteristics between FpHT‐treated and untreated hypogonadal patients undergoing micro‐TESE, stratified by karyotype (46, XY vs. Klinefelter syndrome).

	46, XY			KS	
	Treated	Untreated	*p*	Treated	Untreated	*p*
Total patients, *n* (%)	126 (51.9)	117 (48.1)		175 (4.6)	209 (54.4)	
Age (years), median (IQR)	36 (31–39)	35 (32–39.5)	0.6	29 (17.5–34)	16 (14–27)	**<0.001** ^a^
BMI (kg/m^2^), median (IQR)	29.8 (26.3–32.8)	27.2 (24.8–31.7)	**0.01** ^a^	26.8 (23.5–31.1)	22.6 (19.4–26.3)	**<0.001** ^a^
Smoker, *n* (%)	36 (28.1)	46 (40)	0.1c	59 (33.7)	37 (17.7)	**0.001c**
Total TV (mL), median (IQR)	14 (7–21)	16 (11–25)	**0.013** ^a^	3 (2–4)	4 (2–6)	**0.006** ^a^
**Comorbidities,** *n* **(%)**						
History of undescended testis	30 (23.4)	24 (20.9)	0.21	37 (21.1)	45 (21.5)	0.38^b^
Varicocele	19 (14.8)	12 (10.4)	0.40c	10 (5.7)	12 (5.7)	1.0c
AZFc microdeletions	5 (4)	5 (4.4)	1^b^	0 (0)	0 (0)	—
Inguinal hernia	2 (1.6)	4 (3.5)	0.71^b^	7 (4.0)	5 (2.4)	0.54c
Diabetes mellitus type 1	1 (0.8)	0 (0)	1^b^	0 (0)	1 (0.5)	1.0^b^
Diabetes mellitus type 2	4 (3.1)	2 (1.7)	0.69^b^	1 (0.6)	0 (0)	0.46^b^
Arterial hypertension	8 (6.2)	8 (7)	1c	6 (3.4)	1 (0.5)	**0.05** ^b^
Hypothyreosis	13 (10.2)	7 (6.1)	0.36c	5 (2.9)	4 (1.9)	0.74^b^
**Hormone, median (IQR)**						
FSH (IU/L)	18.15 (10.4–27.1)	20 (12.8–3)	0.24^a^	30.7 (23.0–42.1)	33.0 (24.6–42.6)	0.6^a^
LH (IU/L)	7.15 (4.3–11.3)	6.4 (4.4–10.9)	0.75^a^	14.8 (11.4–20.0)	14.2 (9.6–19.0)	0.08^a^
T (nmol/L)	7.6 (5.9–9)	9.9 (8.7–11.5)	**<0.001** ^a^	6.5 (4.6–7.7)	9.5 (8.0–11.4)	**<0.001** ^a^
fT (pmol/L)	193 (146–233)	222 (186–274.3)	**<0.001** ^a^	135.0 (94.3–172.0)	181.0 (145.0–225.8)	**<0.001** ^a^

*Note*: Percentages for treated and untreated patients refer to the hypogonadal subgroup within each karyotype (46, XY or Klinefelter syndrome).

Abbreviations: BMI, body mass index; FSH, follicle‐stimulating hormone; fT, = free testosterone; KS, Klinefelter syndrome; LH, luteinizing hormone, T, total testosterone; TV, testicular volume.

^a^ Mann–Whitney test.

^b^ Fisher's test.

^c^ Chi‐squared test.

These baseline imbalances were included as covariates in multivariable logistic regression models to reduce confounding when assessing the association between FpHT and SRR in hypogonadal patients.

### 3.3 Influence of FpHT on Hormonal Production

In both cohorts, FpHT led to a significant increase in T production, resulting in higher serum levels (*p* < 0.001), with a mean delta change of +3.1 nmol/L in KS patients and +5.6 nmol/L in those with a normal karyotype. The impact of treatment on hormonal levels is summarized in Table 3. Subclassifications according to the type of therapy administered are reported below.

**TABLE 3:** Changes in hormonal parameters (FSH, LH, testosterone, and free testosterone) following different type of FpHT, stratified by karyotype (46, XY and Klinefelter syndrome).

	46, XY			KS		
	N° treated patients	Delta	*p*	N° treated patients	Delta	*p*
**FSH, median (IQR)**						
All treated	126	1.45 (‐6.3 to 5.5)	0.974	175	−5.4 (‐15.2 to 1.4)	**<0.001**
hCG	28	−9.4 (‐14.7 to ‐5.3)	**<0.001**	121	−9 (‐19.8 to ‐2.4)	**<0.001**
hCG + rFSH	16	−15.1 (‐22.2 to ‐10.2)	**0.002**	16	−10.2 (‐13.8 to ‐5.6)	**0.001**
Anastrozole	59	4.3 (1.5 to 7.2)	**<0.001**	28	3.6 (0.2 to 8.3)	**0.004**
SERMs	20	2.3 (‐0.2 to 7.1)	0.135	10	−5.4 (‐14.5 to 3)	0.313
Other combinations	3	3.9 (1.9 to 6.3)	0.500	—	—	
**LH, median (IQR)**						
All treated	126	0.2 (‐3.3 to 1.8)	0.275	175	−3 (‐7.2 to 0.4)	**<0.001**
hCG	28	−4.8 (‐ 6.6 to ‐2.5)	**<0.001**	121	−4.8 (‐8.1 to ‐1.2)	**<0.001**
hCG + rFSH	16	−6.6 (‐ 9.7 to ‐ 4.1)	**0.002**	16	−6.1 (‐9.2 to ‐ 4.4)	**<0.001**
Anastrozole	59	0.8 (‐0.1 to 2.8)	**<0.001**	28	3.1 (1.8 to 7.4)	**<0.001**
SERMs	20	0.8 (‐1.4 to 2.4)	0.378	10	0.1 (‐8 to 3)	0.641
Other combinations	3	1.2 (1.1 to 1.5)	0.500	—	—	
**Testosterone, median (IQR)**						
All treated	126	5.6 (1.6 to 9.8)	**<0.001**	175	3.1 (0.6 to 5.6)	**<0.001**
hCG	28	3 (0.6 to 7.8)	**<0.001**	121	3.2 (0.5 to 5.8)	**<0.001**
hCG + rFSH	16	13.1 (6.7 to 16.9)	**0.004**	16	4.2 (2.4 to 6.2)	**0.002**
Anastrozole	59	5.8 (2.3 to 9.4)	**<0.001**	28	2.8 (0.6 to 4)	**<0.001**
SERMs	20	6.6 (1 to 15.3)	**0.001**	10	1.6 (‐2.8 to 3.6)	0.641
Other combinations	3	9.7 (‐0.5 to 10.7)	0.500	—	—	
**Free Testosterone, median (IQR)**						
All treated	126	163.5 (34 to 269)	**<0.001**	175	71 (16 to 133)	**<0.001**
hCG	28	35 (3.75 to 129.75)	**0.002**	121	72 (15 to 133)	**<0.001**
hCG + rFSH	16	368.5 (207 to 553)	**0.004**	16	109 (71.5 to 167)	**0.003**
Anastrozole	59	179 (84 to 260)	**<0.001**	28	54.5 (19.2 to 116.2)	**0.001**
SERMs	20	164.5 (‐6.5 to 292.8)	**0.011**	10	0.5 (‐83.5 to 48.2)	0.844
Other combinations	3	150.5 (‐31.5 to 309.8)	0.500	—	—	

*Note*: Delta values represent the median change from baseline to post‐treatment levels. Bold values indicates significant values *p* < 0.001.

### 3.4 Gonadotropins

Exogenous gonadotropins were more frequently used among KS patients (*p* < 0.01): 121 received hCG monotherapy, and 16 received combined treatment with hCG and rFSH. In the 46, XY cohort, 44 patients underwent gonadotropin therapy—28 with hCG alone and 16 with the combined hCG + rFSH regimen. No statistically significant differences were observed in treatment duration between the two groups, with a median of 6 (4–10) months for hCG and 5 (4–10) months for rFSH.

Gonadotropin therapy produced a significant decrease in FSH and LH levels in the treated group (both *p* < 0.001), accompanied by a marked rise in serum T and fT concentrations (both *p* < 0.001).

When hormonal response was further analyzed by treatment regimen—hCG monotherapy versus combined hCG + rFSH—a significant increase in T and fT was consistently observed across both therapeutic schemes and karyotype groups (both *p* < 0.001), with notably greater increases seen in patients with a normal karyotype receiving combined therapy. Despite the marked reductions in FSH, levels remained within the normal range under both treatment regimens, with greater decreases observed with combined therapy compared with monotherapy in both groups (*p* < 0.05).

### 3.5 Aromatase Inhibitors

Aromatase inhibitors were prescribed to 86 patients (59 with a normal karyotype and 28 with KS), with a median treatment duration of 5 months (3.2–6).

Aromatase inhibitor therapy induced significant increases in both FSH and LH levels within the treated group (both *p* < 0.001). Regarding androgens, treatment resulted in a significant rise in T (*p* = 0.003) and fT (*p* = 0.001), confirming a consistent stimulatory effect on endogenous androgen production across both karyotype groups.

### 3.6 SERMs

A total of 30 patients received SERMs (tamoxifen or clomiphene) prior to surgery, including 20 with a normal karyotype and 10 with KS, with a median treatment duration of 3.2 months (IQR 2–5).

SERM therapy did not significantly affect gonadotropin levels in either group (*p* > 0.1 for both LH and FSH). When androgen levels were analyzed by karyotype, contrasting responses were observed: a significant increase in total testosterone (*p* = 0.001) and free testosterone (*p* = 0.01) was detected only among patients with a normal karyotype, whereas in KS patients, the rise in androgen levels did not reach statistical significance for either parameter.

No statistically significant differences in T or fT deltas were observed when comparing gonadotropins, aromatase inhibitors, and SERMs among patients with a normal karyotype. In patients with KS, intergroup comparisons revealed a smaller increase following SERM therapy; however, this finding is likely attributable to the limited sample size.

Overall, significantly greater increases were observed in patients with a normal karyotype compared with those with KS (ΔT: 5.6 vs. 3.1 nmol/L; ΔfT: 163.5 vs. 71.0 pmol/L; both *p* < .001).

### 3.7 Influence of the FpHT on Sperm Retrieval Rate and Spermatogenesis

A comparison of SRR rates and histological patterns was performed between eugonadal and hypogonadal patients, and subsequently between treated and untreated hypogonadal men, stratified by karyotype. The results are summarized in Table 4.

**TABLE 4:** Comparison of histological patterns and SRR among patients, stratified by hormonal category, FpHT, and karyotype.

	Eugonadism	Total hypogonadism	*p*‐value	Untreated hypogonadism	Treated hypogonadism	*p*‐value
**Normal karyotype**	412	243		117	126	
**Histology, *n* (%)**						
Tubular shadows	—	1 (0.4)	—	—	1 (0.8)	—
Sertoli Cell Only Syndrome	169 (41)	103 (42.4)	0.79	51 (43.6)	52 (41.3)	1.00
Maturation arrest	110 (26.7)	65 (26.7)	1.00	28 (24.3)	37 (29.4)	0.51
Hypospermatogenesis	133 (32.3)	74 (30.5)	0.69	38 (33.0)	36 (28.6)	0.49
**Bergmann–Kliesch score, *n* (%)**						
Right						
0	307 (74.5)	187 (77.0)		81 (70.4)	106 (82.8)	
1	3 (0.7)	3 (1.2)		0 (0.0)	3 (2.3)	
2	10 (2.4)	3 (1.2)		2 (1.7)	1 (0.8)	
≥3	24 (5.8)	4 (1.6)		2 (1.7)	2 (1.6)	
NA	68 (16.5)	46 (18.9)		32 (27.4)	14 (11.1)	
Left						
0	307 (74.5)	183 (75.3)		87 (75.7)	96 (75.0)	
1	9 (2.2)	5 (2.1)		2 (1.7)	3 (2.3)	
2	5 (1.2)	1 (0.4)		1 (0.9)	0 (0.0)	
≥3	11 (2.7)	7 (2.9)		6 (5.2)	4 (3.1)	
NA	80 (19.4)	45 (18.5)		20 (17.4)	25 (19.5)	
**Positive sperm retrieval, *n* (%)**	144 (35)	82 (33.7)	0.80	43 (37.4)	39 (30.5)	0.32
**Klinefelter Syndrome**	217	384		209	175	
**Histology, *n* (%)**						
Tubular shadows	2 (0.9)	7 (1.8)	0.50	3 (1.4)	4 (2.3)	0.70
Sertoli Cell Only Syndrome	101 (46.5)	223 (58.1)	**<0.01**	121 (57.9)	102 (58.3)	1.00
Maturation arrest	62 (28.6)	96 (25)	0.39	55 (26.3)	41 (23.4)	0.59
Hypospermatogenesis	52 (24)	58 (15.1)	**<0.01**	30 (14.4)	28 (16.0)	0.76
**Bergmann–Kliesch score (Max)**						
Right						
0	187 (86.2)	354 (92.2)		189 (90.4)	165 (94.3)	
1	0 (0.0)	2 (0.5)		2 (1.0)	0 (0.0)	
2	1 (0.5)	0 (0.0)		0 (0.0)	0 (0.0)	
≥ 3	0 (0.0)	0 (0.0)		0 (0.0)	0 (0.0)	
NA	29 (13.4)	28 (7.3)		18 (8.6)	10 (5.7)	
Left						
0	193 (88.9)	348 (90.6)		191 (91.4)	157 (89.7)	
1	2 (0.9)	1 (0.3)		1 (0.5)	0 (0.0)	
2	1 (0.5)	0 (0.0)		0 (0.0)	0 (0.0)	
≥ 3	0 (0.0)	0 (0.0)		0 (0.0)	0 (0.0)	
NA	21 (9.7)	35 (9.1)		17 (8.1)	18 (10.3)	
**Positive sperm retrieval, *n* (%)**	81 (37.3)	100 (26)	**<0.01**	63 (30.1)	37 (21.1)	0.06

Among 46, XY patients, histological patterns were similarly distributed between hypogonadal and eugonadal men (*p* = 0.40), and SRR did not differ significantly between the two groups (*p* = 0.80). Likewise, within the hypogonadal subgroup, no significant differences in histological findings were observed between patients who received FpHT and those who did not (*p* > 0.50).

SRR remained comparable across baseline testosterone categories: 28.9% for T < 8 nmol/L, 30.3% for T between 8 and 12 nmol/L, and 36.6% for T > 12 nmol/L (*p* = 0.21), data not shown. Preoperative FpHT also did not significantly affect retrieval outcomes, with SRRs of 37.4% in untreated men and 30.5% in those treated (*p* = 0.32).

In contrast, among KS patients, the prevalence of SCO was significantly higher in hypogonadal men compared with their eugonadal counterparts (*p* < 0.01), whereas hypospermatogenesis was less frequent (*p* < 0.01). Consistent with these histological findings, hypogonadal KS patients demonstrated a significantly lower SRR than eugonadal KS men (*p* < 0.01).

SRR also varied significantly according to baseline testosterone levels: 24.3% in patients with T < 8 nmol/L, 30.8% in those with T between 8 and 12 nmol/L, and 35.9% in those with T > 12 nmol/L (*p* = 0.04). Post‐hoc analysis showed a significant difference between patients with T > 12 nmol/L and those with T < 8 nmol/L (*p* = 0.016), whereas differences between intermediate groups were not significant (both *p* = 1.00), data not shown.

Within the hypogonadal KS subgroup, SRR was 30.1% in untreated patients and 21.1% in those receiving FpHT, a difference that approached statistical significance (*p* = 0.06).

In both patient groups, the median Bergmann–Kliesch score was 0 for both testes. The distribution of Bergmann–Kliesch scores is presented in Table 4.

### 3.8 Multivariate Logistic Regression

To account for baseline imbalances between groups and potential confounding, multivariate logistic regression models were fitted in the overall 46, XY and KS cohorts and subsequently repeated in the hypogonadal subgroup only. The models included age, BMI, testicular volume, major comorbidities, smoking status, baseline and post‐treatment hormonal levels, FpHT exposure, and the FSHB‐211 polymorphism as covariates.

**FIGURE 2:**
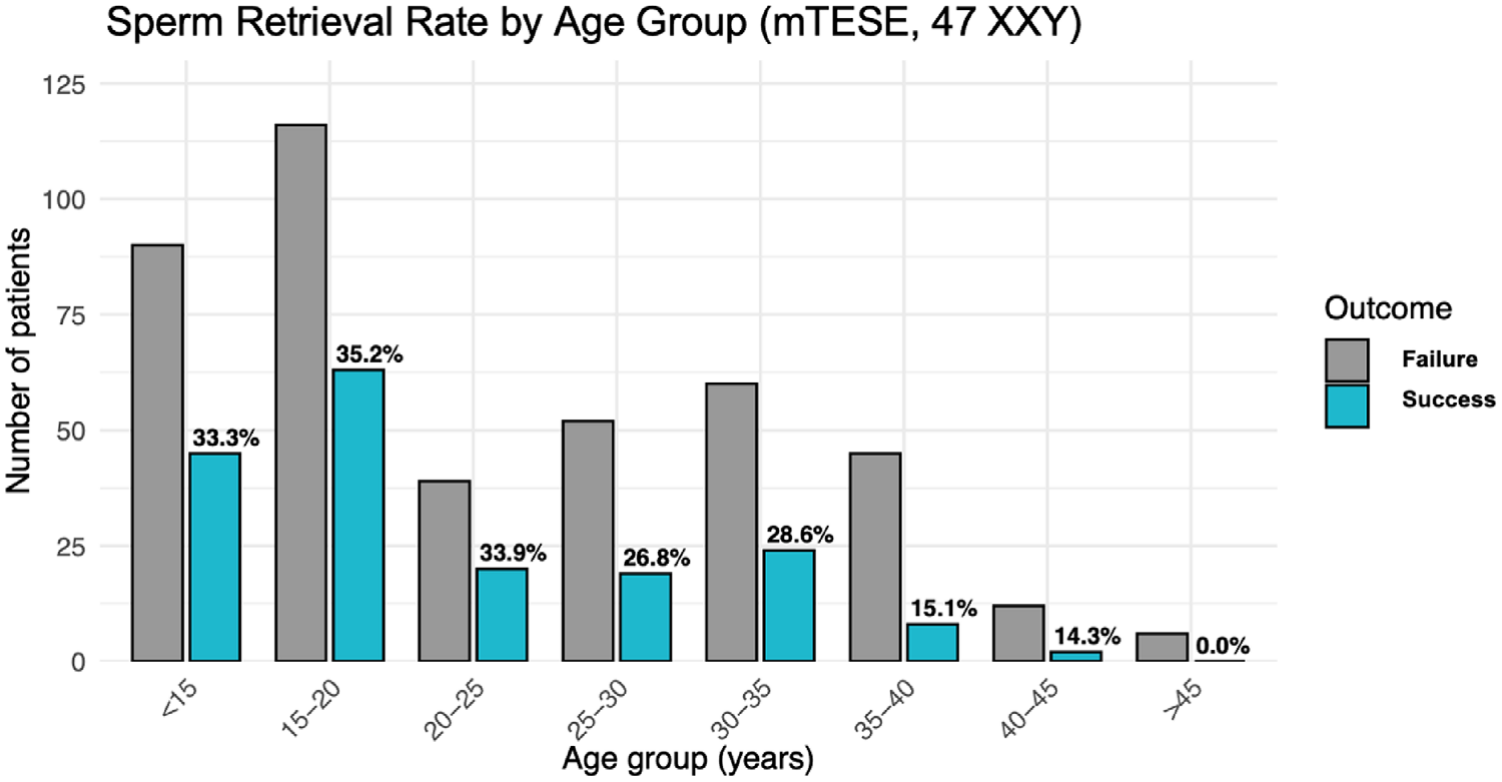
Patients with Klinefelter syndrome undergoing mTESE: Success rate by age group.

In the 46, XY cohort, none of the variables showed a statistically significant association with SRR, although FSH levels approached significance (OR 0.98, 95% CI 0.96–1.00; *p* = 0.056). Among KS patients, younger age emerged as the only independent predictor of successful sperm retrieval, with increasing age being significantly associated with a lower likelihood of sperm finding (OR 0.97, 95% CI 0.94–0.99; *p* = 0.011). This effect remained consistent in the hypogonadal subgroup (OR 0.97, 95% CI 0.93–1.00; *p* = 0.038), as shown in Figure 2. No other variables were significantly associated with SRR.

### 3.9 AZFc

Among the 31 patients with AZFc microdeletion, 21 (67.7%) were eugonadal and 10 (32.3%) hypogonadal. SRR were similar between the two groups (33% vs. 40%; *p* = 1.0). Within the hypogonadal subgroup, SRR was identical in treated and untreated patients (both 40%; *p* = 1.0), indicating no significant impact of hypogonadism or hormonal therapy on retrieval outcomes.

### 3.10 Hypogonadism: Hypergonadotropic (Primary) Versus Normogonadotropic (Functional)

In subgroup analysis on patients with normal karyotype, men with NOA and primary hypogonadism showed significantly smaller testicular volume compared with those with functional hypogonadism (14 mL vs. 19.5 mL; *p* < 0.01), while comorbidity rates were comparable. Free testosterone levels were lower in the primary hypogonadism group both before and after therapy (*p* < 0.02), whereas total testosterone remained similar.

## 4 Discussion

This study represents our contribution to clarifying the efficacy of preoperative FpHT in cases of NOA associated with hypogonadism, evaluating its impact on endogenous androgenic production, spermatogenesis, and SRR, based on a highly selected cohort of men. The results of our analyses show that preoperative FpHT restores androgen levels but does not improve spermatogenic activity or sperm retrieval during mTESE in men with NOA, independently from the gonadotropin level or the presence of AZFc microdeletion.

Different authors have proposed a correlation between T and SRR, with hypogonadism emerging as a predictive variable for surgical outcomes, suggesting that reduced baseline androgen levels are associated with worse SRR [19] and that hormonal optimization could improve SRR. Schlegel's group was the first to show this effect also in patients with KS [39].

We could show in a previously published cohort of patients with KS that baseline serum T values < 7.5 nmol/L were indicators of worse SRR, especially in the presence of LH levels > 17.5 IU/L and age > 25 years, highlighting the possible role of T as a preoperative predictive value [24]. These results were also confirmed by Achermann et al., who reported worse surgical outcomes for hypogonadal individuals in a mixed cohort of patients with NOA undergoing mTESE, especially when associated with elevated FSH values, once again emphasizing the possible preoperative predictive role of these values [19].

As an attempt to improve spermatogenesis through increased T production [20, 40], FpHT is used in reproductive andrology primarily in the preoperative treatment of various forms of hypogonadism associated with NOA, although its application extends to a broader spectrum of male infertility scenarios [20, 41]. With the Aphrodite criteria [20], Esteves et al. accurately defined which categories of patients may potentially benefit from FpHT treatment, and could highlight that in men with hypogonadal NOA, the benefit of FpHT varies based on the gonadotropic profile. According to these criteria, in patients with normogonadotropic NOA, hormonal therapy can lead to multiparametric improvement: increased serum T, histological progression of spermatogenesis, and increased SRR. Conversely, in patients with hypergonadotropic NOA, efficacy is limited to normalization of androgenic levels, suggesting that testicular damage is already too advanced to influence spermatogenic capacity. However, the evidence remains controversial. Shinjo et al. demonstrated opposite results in hypergonadotropic NOA patients undergoing repeat mTESE following initial retrieval failure. In their cohort of 25 men, gonadotropin pretreatment increased intratesticular testosterone (ITT) levels without corresponding changes in serum T, improved testicular histology from maturation arrest to hypospermatogenesis, and raised SRR from 0% to 15% [42]. These data suggest that FpHT may retain a role in carefully selected subgroups, even within hypergonadotropic forms.

In our study we found different results depending on the karyotype analyzed. In euploid subjects, baseline T values do not appear to correlate with surgical outcomes, with no difference emerging in terms of SRR and histological analysis between eugonadal and hypogonadal men, either in direct comparison between classes or when stratifying patients based on baseline serum T levels. Regression analyses did not demonstrate any influence of baseline serum T on SRR, instead suggesting a possible—though non‐significant—negative effect of increasing FSH levels on SRR.

Conversely, in patients with KS, the presence of hypogonadism was found to be associated with worse surgical and histological outcomes, especially for baseline T values < 8 nmol/L, with reduced SRR and higher prevalence of SCO compared to eugonadal individuals. In this subgroup, regression analyses again failed to demonstrate an effect of baseline serum T on SRR, showing instead that age was the only factor significantly associated with SRR, with a progressive decline in retrieval probability over time and a marked reduction beyond 26–27 years. Both results are consistent with the findings previously reported by Rohayem et al. [24].

Given these findings on baseline T as a predictor, we next examined whether active hormonal optimization could actually improve surgical outcomes. Our cohort was predominantly composed of patients with NOA and hypogonadism due to primary testicular failure. In this population, despite preoperative hormonal treatment, the effect of the FpHT proved to be limited only to an improvement in serum androgenic levels, with significant increases in T and fT in treated patients and regardless of karyotype, although the magnitude of this increase was lower in individuals with KS. Critically, we did not observe improvements in either histological progression or SRR, not even when analyzing specific subgroups such as patients with normogonadotropic NOA and hypogonadism (*n* = 22, all with normal karyotype).

Busch et al. reported significant differences in SRR according to FSH‐211 polymorphism type (GG, GT, TT) [25, 43], with poorer outcomes in TT polymorphism carriers, suggesting a potential positive role for exogenous gonadotropin therapy (especially rFSH) in these patients. However, in our study we observed no differences in SRR according to polymorphism type, nor any influence in regression analysis. This is likely due to differences in the analyzed cohorts, as ours consisted of highly selected individuals, in whom, particularly considering only those with normal karyotype, genetic alterations may represent the primary cause of infertility and therefore may not respond to gonadotropin therapy.

The hypothesis correlating reduced T level with poor spermatogenic capacity is supported by the physiological role of T during spermatogenesis, which, after being produced by Leydig cells (LC) following LH stimulation, acts in its ITT together with FSH, modulating the activity of Sertoli cells (SC) through autocrine, endocrine, and paracrine pathways [42, 43, 44, 45, 46].

Although the underlying mechanisms remain only partially understood, studies based on the suppression of T production through gonadotropin blockade have observed that reductions in ITT levels are associated with impairment of spermatogenesis [20, 21, 22]. In humans, however, it remains unclear what the minimum ITT levels necessary for maintaining the spermatogenic process are [42], given that its intratesticular concentrations are approximately 80–100 times higher than serum levels and considering the limited literature on the subject and the difficulty of its measurements [42, 47].

The lack of improvement in spermatogenesis and SRR despite the increase in serum T suggests several possible explanations. Firstly, the ITT levels necessary to sustain spermatogenesis may have already been sufficient at baseline, rendering the further increase in serum T inconsequential for the spermatogenic process. This underscores that serum T is not a reliable surrogate marker for ITT. Secondly, in patients with normal karyotype—a highly selected cohort of idiopathic NOA—the spermatogenic deficit is probably attributable to genetic or molecular causes not correctable through hormonal optimization. Finally, in patients with KS, testicular damage may already be too advanced, with a quantitative reduction in areas of parenchyma with residual spermatogenesis and LCs, which also explains the reduced increase in androgen levels compared to eugonadal individuals [48, 49].

It is important to emphasize a relevant methodological aspect: in selecting our cohort, we applied rigorous criteria, including only patients with a Bergmann–Kliesch score < 8 to exclude subclinical obstructive forms [38]. This choice may explain, at least in part, the discrepancy with the results of Esteves et al. [20]. It is plausible that the benefits they reported were amplified by the inadvertent inclusion of patients with a subclinical obstructive component, in whom the improvement in SRR might reflect better patency rather than a real effect of FpHT on spermatogenesis.

### 4.1 Clinical Implications

Based on our findings, FpHT should be repositioned in clinical practice: it remains a valid therapeutic option for treating symptomatic hypogonadism in men with NOA and active reproductive desire, but it should not be routinely employed as a strategy to improve SRR before mTESE. These results align with current EAU guidelines, which do not recommend routine preoperative hormonal therapy due to insufficient evidence of fertility improvement.

The clinical decision to initiate FpHT should therefore be driven primarily by the presence of hypogonadal symptoms rather than by the expectation of enhancing surgical outcomes. In hypogonadal men scheduled for mTESE, FpHT may be considered to optimize metabolic and sexual health, improve quality of life. However, the impact on clinically significant parameters—such as quality of life (QoL), sexual function (IIEF‐5), and hypogonadal symptomatology (HIS)—was not systematically evaluated in the present study and represents an objective for future research.

The wide variability in SRR observed across the entire range of FSH values confirms that this hormonal parameter cannot be considered a reliable preoperative predictor, in line with what has emerged from the most recent meta‐analyses [26, 27].

In contrast to FSH, age emerged as a significant predictor in patients with KS, with a progressive decline in the probability of retrieval with increasing years and a marked reduction beyond 26–27 years. These results highlight how these patients could potentially benefit from early mTESE at a young age, ideally after puberty (≥16 years), to maximize the chances of cryopreserving sperm before progressive testicular deterioration, thus preserving fertility in view of possible future reproductive desires. However, the usefulness of age as a predictive parameter in clinical practice remains controversial. Despite statistical significance, low sensitivity and specificity limit its decisional value: positive sperm retrievals were obtained even in patients significantly beyond the 26–27‐year cut‐off, making the application of rigid thresholds problematic.

### 4.2 Strengths and Limitations

This study presents several notable strengths. First, we analyzed a relatively large cohort of highly selected NOA patients, applying rigorous inclusion criteria—including a Bergmann–Kliesch score < 8—to exclude subclinical obstructive forms that could have confounded the results. Second, we performed comprehensive subgroup analyses stratified by karyotype and gonadotropin status, allowing for nuanced interpretation across clinically relevant patient categories. Third, our study included structured hormonal follow‐up with systematic pre‐ and post‐treatment assessments. Finally, our cohort reflects real‐world clinical practice from a single tertiary referral center, enhancing external validity.

However, this study is not without limitations. First, its retrospective design and absence of randomization may introduce selection bias and confounding factors. Second, we included patients of younger age (at least Tanner stage 3–4) in whom the spermatogenic process is known to be still under development, which may have influenced the results. Third, although our cohort was large overall, subgroup analyses—particularly those concerning normogonadotropic hypogonadal men—were limited by reduced sample size, reducing statistical power. Fourth, we did not measure ITT levels, which would have provided direct insight into the local androgenic milieu. Fifth, the impact of FpHT on patient‐reported outcomes—such as quality of life, sexual function, and hypogonadal symptomatology—was not assessed. Finally, we did not evaluate long‐term reproductive outcomes, such as live birth rates following mTESE and assisted reproductive techniques. Furthermore, the considerable heterogeneity in study design, patient selection, treatment protocols, and reported outcomes across published studies makes direct comparisons unreliable and further underscores the urgent need for well‐designed prospective trials.

To comprehensively assess not only the effect of specific FpHT regimens on T levels and SRR, but also their impact on hypogonadism‐related symptoms and live birth rates following mTESE, prospective randomized controlled clinical trials are warranted to confirm our findings.

## 5 Conclusion

Preoperative FpHT proved effective in improving serum androgen levels in men with NOA associated with hypogonadism, regardless of karyotype. However, this hormonal enhancement did not translate into higher sperm retrieval rates, suggesting that the beneficial endocrine response induced by FpHT does not necessarily reflect a parallel improvement in spermatogenic potential.

These findings highlight the need to reconsider the current clinical approach in hypogonadal patients with NOA. FpHT should be considered primarily for treating symptomatic hypogonadism rather than as a routine strategy to improve fertility outcomes before mTESE, supporting current EAU guideline recommendations.

## Author Contributions

Simone Bier, Michael Zitzmann, and Mattia Anfosso designed the study. Sabine Kliesch, Jann Frederik Cremers, and Simone Bier were responsible for clinical, histological, and surgical procedures. Mattia Anfosso carried out the data acquisition, analyzed the data and drafted the manuscript. Simone Bier carried out the first revision of the manuscript. The manuscript was then critically revised by Jann‐Frederik Cremers, Maria Schubert, Michael Zitzmann, and Sabine Kliesch. All authors approved the submitted and final version.

## Funding

This research was Funded by the German Federal Ministry of Research, Technology, and Space (BMFTR) as part of the project ReproTrackMS – Centre for Research and Development of Reproductive Scientists (grant no: 01GR2303).

## Conflicts of Interest

The authors declare no conflicts of interest.
